# Development and Utilization of InDel Markers to Identify Peanut (*Arachis hypogaea*) Disease Resistance

**DOI:** 10.3389/fpls.2015.00988

**Published:** 2015-11-13

**Authors:** Lifeng Liu, Phat M. Dang, Charles Y. Chen

**Affiliations:** ^1^Department of Crop, Soil and Environmental Sciences, Auburn UniversityAuburn, AL, USA; ^2^Department of Agronomy, Agricultural University of HebeiBaoding, China; ^3^National Peanut Research Laboratory, United States Department of Agriculture-Agricultural Research ServiceDawson, GA, USA

**Keywords:** InDel markers, cultivated peanut, genetic diversity, disease resistances

## Abstract

Peanut diseases, such as leaf spot and spotted wilt caused by Tomato Spotted Wilt Virus, can significantly reduce yield and quality. Application of marker assisted plant breeding requires the development and validation of different types of DNA molecular markers. Nearly 10,000 SSR-based molecular markers have been identified by various research groups around the world, but less than 14.5% showed polymorphism in peanut and only 6.4% have been mapped. Low levels of polymorphism limit the application of marker assisted selection (MAS) in peanut breeding programs. Insertion/deletion (InDel) markers have been reported to be more polymorphic than SSRs in some crops. The goals of this study were to identify novel InDel markers and to evaluate the potential use in peanut breeding. Forty-eight InDel markers were developed from conserved sequences of functional genes and tested in a diverse panel of 118 accessions covering six botanical types of cultivated peanut, of which 104 were from the U.S. mini-core. Results showed that 16 InDel markers were polymorphic with polymorphic information content (PIC) among InDels ranged from 0.017 to 0.660. With respect to botanical types, PICs varied from 0.176 for *fastigiata* var., 0.181 for *hypogaea* var., 0.306 for *vulgaris* var., 0.534 for *aequatoriana* var., 0.556 for *peruviana* var., to 0.660 for *hirsuta* var., implying that *aequatoriana* var., *peruviana* var., and *hirsuta* var. have higher genetic diversity than the other types and provide a basis for gene functional studies. Single marker analysis was conducted to associate specific marker to disease resistant traits. Five InDels from functional genes were identified to be significantly correlated to tomato spotted wilt virus (TSWV) infection and leaf spot, and these novel markers will be utilized to identify disease resistant genotype in breeding populations.

## Introduction

Various types of molecular markers, such as random amplified polymorphic DNA (RAPD) (Williams et al., 1990; Burow et al., 1996; Subramanian et al., 2000); amplified fragment length polymorphism (AFLP) (Vos et al., 1995; He and Prakash, 1997); inter simple sequence repeat (ISSR) markers (Zietkiewicz et al., 1994; Raina et al., 2001) and simple sequence repeats (SSR) (Tautz, 1989; Liang et al., 2009), have been used in detecting the genetic diversity of plant germplasm resources (Cuc et al., 2008; Jiang et al., 2010; Moretzsohn et al., 2013), construction of genetic linkage maps (Varshney et al., 2009; Hong et al., 2010; Gautami et al., 2012; Nagy et al., 2012; Qin et al., 2012; Shirasawa et al., 2013), molecular marker-assisted selection (MAS) and mapping and cloning of genes/QTL (Chu et al., 2011; Ravi et al., 2011; Sujay et al., 2012) in peanut. Microsatellite or simple sequence repeat (SSR) markers have been developed using sequences derived from SSR-enriched genomic libraries and expressed sequence tags (ESTs) (Guo et al., 2009; Koilkonda et al., 2012; Wang et al., 2012; Zhang et al., 2012) and have been utilized to investigate genetic diversity for the US peanut mini-core collection (Belamkar et al., 2011; Wang et al., 2011; Chen et al., 2014), Chinese peanut mini-core collection (Jiang et al., 2010, 2014), and ICRISAT peanut mini-core collections (Ren et al., 2010; Mukri et al., 2012; Upadhyaya et al., 2012). The functional SNP markers from *FAD2A*/*FAD2B* genes have been used to screen the U.S. mini-core collection (Wang et al., 2013). Another new kind of marker called Start codon targeted polymorphism (SCoT) was also developed and showed the potential use for studying the genetic diversity and relationship in cultivated peanut (Xiong et al., 2011). Approximately 10,000 molecular markers have been identified by various research groups around the world, but only 14.5% showed polymorphism in peanut and only 6.4% were mapped (Zhao et al., 2012), mainly due to the fact that cultivated peanut possesses an extremely narrow genetic basis (Xiong et al., 2011). Low genetic diversity among cultivated peanut accessions is likely due to the single hybridization event between two ancient diploid species, likely *Arachis duranensis* (A genome) and *Arachis ipaensis* (B genome) (Burow et al., 2009; Nagy et al., 2012; Shirasawa et al., 2013). Low level of polymorphism limits the application of molecular markers in peanut breeding and genetics studies.

InDels have been recognized as an abundant source of genetic markers that are widely spread across the genome, and there is an increasing focus on polymorphisms of the type short insertions and deletions (InDels) in genomic and breeding research (Lv et al., 2013; Yamaki et al., 2013). Short sequence and homonucleotide repeats tend to accumulate InDels due to polymerase slippage during replication and frame shift InDels in coding regions can result loss of function or non-sense mutation (Rockah-Shmuel et al., 2013). It has been reported that insertions and deletions (InDels) markers were more polymorphic than SSRs in some crops (Liu et al., 2013; Wu et al., 2014). No research of InDel marker in peanut has been reported for trait association. Therefore, it is vital to develop InDel markers in peanut and to apply these markers to associate important traits, such as disease resistance. The objectives of this research were: (1) to develop the gene-specific InDel markers; (2) to evaluate the potential use in genetic diversity study for cultivated peanut; and (3) to identify novel InDel markers that related to the disease-resistant traits.

## Materials and methods

### Plant materials and phenotyping of TSWV and leaf spot

One hundred and eighteen peanut accessions from the USDA peanut germplasm collection in Griffin, GA were used in the study, in which 104 accessions were selected from the US peanut mini-core collection and an additional 14 accessions were selected to represent two botanical types (*hirsuta* var. and *aequatoriana* var.) of cultivated peanut that are not present in the mini-core (Table 1). Twenty seed of each 118 *Arachis hypogaea* accessions were planted at Dawson, GA (31°45′ latitude, −84°30′ longitude) in 2010, 2012, and 2013 under irrigated conditions. The genotypes were planted in two-row plots 3 m long and 0.91 m between rows at a seeding rate of 3 seed m^−1^ in early May with three replications. Before planting, the field area was cultivated and irrigated with 15 mm of water to ensure adequate moisture for uniform seed germination. Crop management for all entries was according to best management practices for soil nutrients, herbicides, and pesticides. For evaluation of TSWV resistance, all plots of each PI were visually rated immediately prior to digging for foliar symptoms on a percentage basis, similar to the 1–10 method described by Tillman et al. (2007) where 1 = no disease and 10 = all plants severely diseased. Disease evaluations for leaf spot resistance were conducted in the field under a reduced fungicide-treatment with one application of 1.5 pt/A chlorothalonil in 2010 and no fungicide application in 2012 and 2013. Plants were rated using the Florida leaf spot scoring system during flowering, 2 weeks before harvest, and immediately prior to harvest (Chiteka et al., 1988). The data was analyzed using SAS Institute (version 9.2, 2009) with PROC GLM under the general linear model. Means were separated using Fisher's Protected LSD at *p* < 0.05.

**Table 1:** **One hundred eighteen accessions from six botanical varieties of cultivated peanuts used for disease evaluation and the InDel marker analysis**.

**Code**	**PI Number**	**Botanical variety**	**Origin**	**Code**	**PI Number**	**Botanical variety**	**Origin**
G001	PI 152146	*fastigiata*	Uruguay	G060	PI 372305	*hypogaea*	Nigeria
G002	PI 155107	*vulgaris*	Uruguay	G061	PI 399581	*hypogaea*	Nigeria
G003	PI 157542	*vulgaris*	China	G062	PI 403813	*vulgaris*	Argentina
G004	PI 158854	*fastigiata*	China	G063	PI 407667	*vulgaris*	Thailand
G005	PI 159786	*hypogaea*	Senegal	G064	PI 429420	*fastigiata*	Zimbabwe
G006	PI 162655	*hypogaea*	Uruguay	G065	PI 442768	*hypogaea*	Zimbabwe
G007	PI 162857	*hypogaea*	Sudan	G066	PI 461434	*hypogaea*	China
G008	PI 196622	*hypogaea*	Cote D'Ivoire	G067	PI 471952	*hypogaea*	Zimbabwe
G009	PI 196635	*hypogaea*	Madagascar	G068	PI 471954	*fastigiata*	Zimbabwe
G010	PI 200441	*fastigiata*	Japan	G069	PI 476432	*hypogaea*	Nigeria
G011	PI 240560	*hypogaea*	South Africa	G070	PI 476636	*hypogaea*	Nigeria
G012	PI 259617	*fastigiata*	Cuba	G071	PI 478819	*vulgaris*	India
G013	PI 259658	*hypogaea*	Cuba	G072	PI 478850	*fastigiata*	Uganda
G014	PI 259836	*fastigiata*	Malawi	G073	PI 481795	*hypogaea*	Zambezia
G015	PI 259851	*hypogaea*	Malawi	G074	PI 482120	*hypogaea*	Zimbabwe
G016	PI 262038	*fastigiata*	Brazil	G075	PI 482189	*fastigiata*	Zimbabwe
G017	PI 268586	*hypogaea*	Zambia	G076	PI 494795	*hypogaea*	Zambia
G018	PI 268696	*hypogaea*	South Africa	G077	PI 496401	*hypogaea*	Burkina
G019	PI 268755	*hypogaea*	Zambia	G078	PI 496448	*hypogaea*	Burkina
G020	PI 268806	*hypogaea*	Zambia	G079	PI 502040	*fastigiata*	Peru
G021	PI 268868	*hypogaea*	Sudan	G080	PI 502111	*peruviana*	Peru
G022	PI 268996	*hypogaea*	Zambia	G081	PI 502120	*peruviana*	Peru
G023	PI 270786	*hypogaea*	Zambia	G082	PI 504614	*hypogaea*	Colombia
G024	PI 270905	*hypogaea*	Zambia	G083	PI 475863	*fastigiata*	Bolivia
G025	PI 270907	*hypogaea*	Zambia	G084	PI 475918	*fastigiata*	Bolivia
G026	PI 270998	*vulgaris*	Zambia	G085	PI 476025	*fastigiata*	Peru
G027	PI 271019	*vulgaris*	Zambia	G086	PI 493329	*fastigiata*	Argentina
G028	PI 274193	*hypogaea*	Bolivia	G087	PI 493356	*fastigiata*	Argentina
G029	PI 288146	*vulgaris*	India	G088	PI 493547	*fastigiata*	Argentina
G030	PI 290536	*hypogaea*	India	G089	PI 493581	*fastigiata*	Argentina
G031	PI 290560	*vulgaris*	India	G090	PI 493631	*fastigiata*	Argentina
G032	PI 290566	*fastigiata*	India	G091	PI 493693	*fastigiata*	Argentina
G033	PI 290594	*hypogaea*	India	G092	PI 493717	*fastigiata*	Argentina
G034	PI 290620	*fastigiata*	Argentina	G093	PI 493729	*fastigiata*	Argentina
G035	PI 292950	*hypogaea*	South Africa	G094	PI 493880	*fastigiata*	Argentina
G036	PI 295250	*hypogaea*	Israel	G095	PI 493938	*fastigiata*	Argentina
G037	PI 295309	*hypogaea*	Israel	G096	PI 497517	*fastigiata*	Brazil
G038	PI 295730	*fastigiata*	India	G097	PI 497639	*fastigiata*	Ecuador
G039	PI 296550	*hypogaea*	Israel	G098	PI 497318	*hypogaea*	Bolivia
G040	PI 296558	*hypogaea*	Israel	G099	PI 497395	*hypogaea*	Bolivia
G041	PI 298854	*hypogaea*	South Africa	G100	PI 494018	*vulgaris*	Argentina
G042	PI 313129	*fastigiata*	Taiwan	G101	PI 494034	*vulgaris*	Argentina
G043	PI 319768	*hypogaea*	Israel	G102	PI 288210	*vulgaris*	India
G044	PI 323268	*hypogaea*	Pakistan	G103	PI 371521	*hypogaea*	Israel
G045	PI 325943	*hypogaea*	Venezuela	G104	PI 461427	*hypogaea*	China
G046	PI 331297	*hypogaea*	Argentina	G105	PI 576613	*hirsuta*	Mexico
G047	PI 331314	*hypogaea*	Argentina	G106	Grif 14051	*aequatoriana*	Guatemala
G048	PI 337293	*hypogaea*	Brazil	G107	PI 576634	*hirsuta*	Mexico
G049	PI 337399	*hypogaea*	Morocco	G108	PI 648241	*hirsuta*	Ecuador
G050	PI 337406	*fastigiata*	Paraguay	G109	PI 648250	*aequatoriana*	Ecuador
G051	PI 338338	*peruviana*	Venezuela	G110	PI 576616	*hirsuta*	Mexico
G052	PI 339960	*fastigiata*	Argentina	G111	PI 648249	*aequatoriana*	Ecuador
G053	PI 343384	*hypogaea*	Israel	G112	PI 648242	*aequatoriana*	Ecuador
G054	PI 343398	*fastigiata*	Israel	G113	PI 648245	*aequatoriana*	Ecuador
G055	PI 355268	*hypogaea*	Mexico	G114	Grif 12579	*aequatoriana*	Ecuador
G056	PI 355271	*hypogaea*	Mexico	G115	PI 576614	*hirsuta*	Mexico
G057	PI 356004	*fastigiata*	Argentina	G116	Grif 12545	*aequatoriana*	Ecuador
G058	PI 370331	*hypogaea*	Israel	G117	PI 576636	*hirsuta*	Mexico
G059	PI 372271	*hypogaea*	Unknown	G118	PI 576637	*hirsuta*	Mexico

### Identification of InDels and primer design

**Figure 1:**
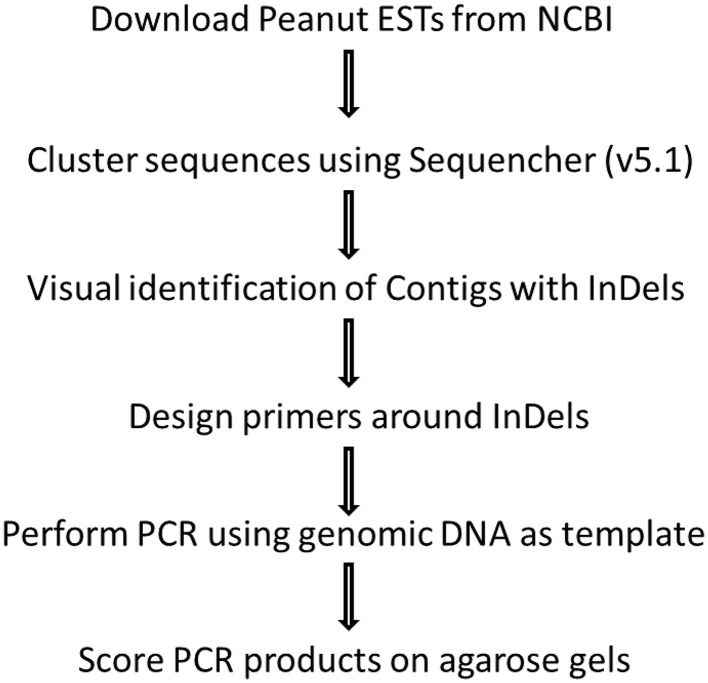
**Flowchart showing identification of peanut EST InDels, primer design, and marker scoring**.

Publically available peanut expressed sequence tags (ESTs) derived from various tissues, developmental stages, and under different biotic and abiotic stresses (Feng et al., 2012) were utilized to identify potential InDel markers. Sequences were downloaded and alignment was performed by Sequencher v5.1 (Gene Codes, Ann Arbor, MI). Individual clusters or contigs were visually observed to identify potential InDels and selected contigs were reassembled using “large gap” criteria for assembly algorithm, resulting in the identification of 48 InDels. Primers were designed using Primer Express 3.0 (Applied Biosystems, Foster City, CA) for the sizes of 150–500 bp. Potential plant gene function was identified through BLASTx (NCBI) and comparison of the sequences according to conserved sequences of functional genes. The procedure of identification of peanut EST InDels, primer design and marker scoring was illustrated by flowchart (Figure 1).

### DNA extraction and PCR

Genomic DNA extraction from dry seeds was performed following the method of Dang and Chen (2013). A Nano-Drop 2000c spectrophotometer (Nano Drop Technologies, USA) was used to evaluate the quality and concentration of all DNA. DNA samples were diluted to 20 ng/μL and PCR conditions were applied: 94°C for 1 min, 30 cycles of 30 s at 94°C, 50°C for 1.0 min, 72°C for 1.5 min, and 1 cycle at 72°C for 10 min. PCR products and DNA molecular weight marker (Promega, Madison, WI) were separated on a 1.2% TAE-agarose gel and image was captured on a Gel Logic 200 Imaging System (Kodak, Rochester, NY).

### Data analysis

Polymorphism Information Content (PIC) based on allelic frequencies among 118 genotypes was calculated for each InDel marker using the following formula: PIC = 1-$\sum x_{i}^{2}$ where *x*_*i*_ is the relative frequency of the *i*th allele of the SSR loci. Clustering analyses were performed using SAS (SAS 9.3; SAS Institute, 2009) to calculate the genetic similarity matrices, and a neighbor-joining (NJ) algorithm (Saitou and Nei, 1987) was used to construct a phylogram from a distance matrix using the MEGA4 software (Tamura et al., 2007). Single marker analysis (SMA) method was used for trait-marker analysis (Jansen and Stam, 1994). It was carried out by PROC GLM of SAS (SAS 9.3; SAS Institute, 2009) with the following linear model: *Y*_*iklm*_ = *u* + *E*_*i*_ + *M*_*k*_ + *F*(*M*)_*kl*_ + *E* x *F(M)*_*ikl*_ + *e*_*iklm*_, where *Y*_*iklm*_ is each observed phenotype, *u* is the population mean, *E*_*i*_ is the effect of year (*i* = 1, 2), *M*_*k*_ is the effect of marker genotype (*k* = 1, 2), *F(M)*_*kl*_ is the effect of PIs within marker genotype (*l* = 1, …, 118), *E x F(M)*_*ikl*_ is the interaction between the effect of year and the effect of PIs within marker genotype, and *e*_*iklm*_ is residual error. Threshold for declaring a marker significant was chosen to be marker-wise *p* < 0.0001, which is approximately equal to an experiment-wise *p* < 0.05 in this study based on 16 polymorphic markers.

## Results

### Polymorphic information of the InDel markers and genetic diversity of the different botanical types based on InDel markers

**Figure 2:**
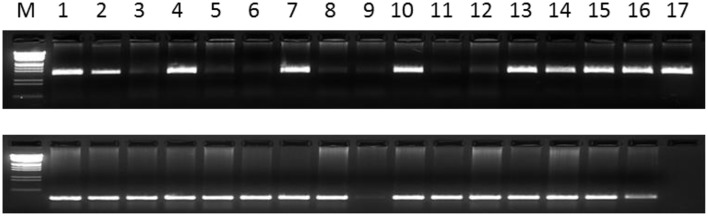
**The fragments amplified by InDel-016 (above) and Indel-042 (bottom)**. The sequences (5′–3′) of Indel-016 primer are TCCTCATCAGGAACTGGGATA(F) and TGCAGCAATAGGACTTCTGG(R). For Indel-042 primer, the sequences (5′–3′) are GGGATTGAGCATGAACGATT(F) and GATAACAAATGGGGGCAAGA(R). 1-PI 152146; 2-PI 155107; 3-PI 157542; 4-PI 158854; 5-PI 159786; 6-PI 162655; 7-PI 162857; 8-PI 196622; 9-PI 196635; 10-PI 200441; 11-PI 240560; 12-PI 259617; 13-PI 259658; 14-PI 259836; 15-PI 259851; 16-PI 262038; 17-PI 268586.

Forty-eight primer-pairs of InDel markers were designed from coding and non-coding regions of the 48 functional genes (Table 2). All 48 primer-pairs generated PCR bands, of which 16 were polymorphic, with different sizes from 200 to 470 bp (Figure 2). The polymorphic information content (PIC) values of each primer ranged from 0.0169 of InDel-03 to 0.5960 of InDel-18 with an average of 0.1349 (Table 3). The distributions of 16 polymorphic InDel markers among the six botanical types were quite different. More polymorphic markers were detected in the botanical types of *hirsuta* var., *aequatoriana* var., *hypogaea* var., and *fastigiata* var. than the other two types of *peruviana* var. and *vulgaris* var. (12, 9, 9, 7, vs. 2, 2) (Table 3). The least polymorphic marker was InDel-03 which only showed in *hirsuta* var., while InDel-16 and InDel-18 showed polymorphism in five of six botanical types. In respect to the different botanical types, PICs varied from 0.176 for *fastigiata* var., 0.181 for *hypogaea* var., 0.306 for *vulgaris* var., 0.534 for *aequatoriana* var., 0.556 for *peruviana* var., to 0.660 for *hirsuta* var., which implied that *hirsuta* var., *peruviana* var., and *aequatoriana* var. have higher genetic diversity than the other types (Table 4).

**Table 2:** **The sequence and annotations of the 48 InDel markers that were developed and used in this study**.

**InDels Primer**	**Sequence from 5′ to 3′**	**Contig**	**Annotation**	**bp difference**	**Location**
Indel-001- F	AATTCGAGGGTGCTGAAATG	[0016]	Metallothionein, type 2	6 bp	3′ non-coding
Indel-001-R	TCAAGGATGCAGCAAGACAC				
Indel-002_F	GCTCAACCGGTTCCAGAATA	[0023]	Allergen II	5 bp	3′ non-coding
Indel-002_R	AGGCAATGCCATAAAAGCAC				
Indel-003_F	GGCCCATGACAAAAGGACTA	[0031]	Peroxidase	6 bp	3′ non-coding
Indel-003_R	GAACTGTGACTGCCACGCAC				
Indel-004_F	GCCTGTAACTGCCTCAAAGC	[0038]	LTP	18 bp	3′ non-coding
Indel-004_R	CATACAAAGACTACAAGAGGARAGG				
Indel-005_F	CAAGCCAGGCTATTGACTCC	[0041]	Isoprene synthase	3 bp	Coding
Indel-005_R	TCGTGAAATGACCATCATTG				
Indel-006_F	AGCTTAACGGCATCCTCTCA	[0055]	Glyceraldehyde-3-phosphate dehydrogenase	10 bp	3′ non-coding
Indel-006_R	GCTTAACAAGTGTAGTGGTAATAGTAG				
Indel-007_F	ACCGTGCTGTGACAAATTCA	[0047]	Hyoscyamine-6-dioxygenase	22 bp	3′ non-coding
Indel-007_R	GCACCTCTACATGAAGGTGAAC				
Indel-008_F	ACGTCTGACCCATGAAATCC	[0061]	Catalase	30 bp	3′ non-coding
Indel-008_R	CGTACACGCGGACAGATTTAG				
Indel-009_F	GCCTTATCAACYCTTTCACCCTC	[0057]	Gibberellin 2-oxidase	15 bp	5′ coding
Indel-009_R	AGCGGCAAGGAGAAGAATTT				
Indel-010_F	AGAGCATTAAGGAGAAAGCTGC	[0100]	LEA 4	3 bp	Coding
Indel-010_R	ATGTTGTCCGGTTGTGGAAT				
Indel-011_F	CTGCAAATTCGACAAGAGCA	[0059]	Cysteine proteinase	5 bp	3′ non-coding
Indel-011_R	GCAGAACATTTCACAGCATACATG				
Indel-012_F	CACATAGTGGGGCCTGATCT	[0113]	1-Cys peroxiredoxin	3 bp	3′ non-coding
Indel-012_R	AACCATATTTAGATTTGTGAGATAGC				
Indel-013_F	CCACCCCCAGAGTACATCAC	[0110]	Vacuolar processing enzyme	69 bp	Coding
Indel-013_R	GATGGATGCAGGATCGAAGC				
Indel-014_F	GGCACAGAGCAAAGTGAACA	[0115]	F-box protein	3 bp	Coding
Indel-014_R	TTCTCAGAACCCCACAAAGG				
Indel-015_F	AGAGAAGCTGTGGGATGACG	[0276]	Auxin repressed protein	2 bp	3′ non-coding
Indel-015_R	CCACAGACCAAACAAGCAGA				
Indel-016_F	TCCTCATCAGGAACTGGGATA	[0160]	Alkaline alpha galactosidase	19 bp	3′ non-coding
Indel-016_R	TGCAGCAATAGGACTTCTGG				
Indel-017_F	GTGGAGGAGTGTACGGAGGA	[0137]	Drought induced protein	7 bp	3′ non-coding
Indel-017_R	CACACAAGAATGAAAGTGTAAAACC				
Indel-018_F	AGCTGGAAAGCAAGAGCAAG	[0177]	Arachin Ahy-3	12 bp	Coding
Indel-018_R	GCTGTTTGCGTTCATGTTGT				
Indel-019_F	CACCGACAACCTAGGCGTAT	[0285]	Lipid binding protein	26 bp	3′ non-coding
Indel-019_R	GAGCAATAGTGACCTTGCATTG				
Indel-020_F	CATTTTCAAACATTACACTCACTCATC	[0294]	Plant lipid transfer protein	5 bp	3′ non-coding
Indel-020_R	CAACACATGCAATGCAACAA				
Indel-021_F	CCGATTCCTTCAGATAGCAC	[0296]	40S ribosomal protein	2 bp	3′ non-coding
Indel-021_R	GAGAAAATTGAAATTCAACTTCATC				
Indel-022_F	GCGGTGAAATCAACTCATCA	[0315]	Cell wall N rich protein	6 bp	Coding
Indel-022_R	CTTTGTTGAAGCCACCGTTG				
Indel-023_F	CATCCGACATGTTACAATACTGAG	[0326]	bZip Transcription factor	26 bp	3′ non-coding
Indel-023_R	CCATTGATAGAGTGATTACAATTTCTC				
Indel-024_F	GTTGTGTTGATCCTTTCATTCGG	[0421]	Glutamate binding	12 bp	5′ non-coding
Indel-024_R	AGACGGTGATGGAGGATACG				
Indel-025_F	GACTCCATAATCGGAATCCAAG	[0495]	Vesicle membrane protein	18 bp	5′ non-coding
Indel-025_R	GCTTGAGCGCTGGAAGTAAC				
Indel-026_F	TCGGCTTACTCTCCCCTGAAC	[0500]	Plastic protein	3 bp	Coding
Indel-026_R	GTCAATCTCGCACCCAAATC				
Indel-027_F	GGCTATTGCAGGTGGAACAC	[0518]	Wound induced protein	3 bp	Coding
Indel-027_R	GACCCCACGTGCTCAAATAC				
Indel-028_F	ACCAATGCATGTGGATCATGC	[0534]	Lipid binding protein	3 bp	5′ non-coding
Indel-028_R	GCAGTGCACAAACAAAGTGC				
Indel-029_F	TTCCTTTGCTTTCCACCATT	[1556]	Protease inhibitor	5 bp	3′ non-coding
Indel-029_R	GCATGATGAGGATTAAAAGATGATAG				
Indel-030_F	TTGAAGGCAGAGGAGGTAGC	[0522]	Remorin	11 bp	3′ non-coding
Indel-030_R	GAAAGGAACATTGAACTAAATTTTGC				
Indel-031_F	CGTCATATCCATCACCACCA	[0581]	Proline rich cell wall protein	12 bp	Coding
Indel-031_R	GGAGGAGTCATGCCACAAGT				
Indel-032_F	AGGAGCAACCGGACACATAC	[0628]	Electron transporter/metal ion	7 bp	3′ non-coding
Indel-032_R	TGCACCTCATCAACCTCTCA				
Indel-033_F	CCTTTAGGCCCAAGGATTTC	[3275]	Salt tolerance protein	3 bp	Coding
Indel-033_R	TGCCTCTAAGTCCCTTCTTATTG				
Indel-034_F	TGCAGCACGTAAGGATCAAG	[0898]	Unknown	3 bp	3′ non-coding
Indel-034_R	TTTGTAACGCAACCTTGCAC				
Indel-035_F	CGTGGGAGGGACAGAGATTA	[1457]	Arginine/serine splicing factor	3 bp	3′ non-coding
Indel-035_R	AGATCGTCCATCACGGCTAC				
Indel-036_F	ATTGGCTTGTGAAGCATTCC	[2962]	ATARLA, GTP binding	3 bp	3′ non-coding
Indel-036_R	CAGCTACATCAACAATGACATGA				
Indel-037_F	CACCCCAAGTTTGGAAAATG	[3189]	Unknown	7 bp	3′ non-coding
Indel-037_R	CACTTGATTGCAAGCTTGTACAAAT				
Indel-038_F	TGAAGTCAGTGACAGTGGTGAA	[3291]	Glycine dehydrogenase	1 bp	3′ non-coding
Indel-038_R	GCAGTCAAAGCACAAGACAAG				
Indel-039_F	ACTTCCAATTCCCAGCACAG	[3482]	Unknown	6 bp	5′ non-coding
Indel-039_R	CCCAATGAAAGCTTGAAGGA				
Indel-040_F	CTTAATAATTTGGATGAAGGATCATC	[3624]	Unknown	6 bp	5′ non-coding
Indel-040_R	CGGTGGTTCCAAAAAGAAGA				
Indel-041_F	AAGCTGCTGAGAGGGAAAGAC	[3694]	Unknown	18 bp	5′ non-coding
Indel-041_R	GCCCACACATGCATAGACAG				
Indel-042_F	GGGATTGAGCATGAACGATT	[3863]	Dihydroxy-acid dehydratase	2 bp	3′ non-coding
Indel-042_R	GATAACAAATGGGGGCAAGA				
Indel-043_F	GATATAGCACCAGCAGCATAGTTTC	[1258]	Unknown	9 bp	3′ non-coding
Indel-043_R	TTTTCAGTCAAATGATGGAAGC				
Indel-044_F	TTGAGGCCCTAAGAATGAGC	[2367]	Cyclin-dependent protein kinase	12 bp	3′ non-coding
Indel-044_R	TTTTTGTCCTCATGAAGAACTACG				
Indel-045_F	GAGGAGGCCAAGAAGGAGTT	[3274]	Frutose-bisphosphate aldolase	2 bp	3′ non-coding
Indel-045_R	TGGCTCCTAACTTATGGCAAA				
Indel-046_F	TGAACTCGAGCGAACATCAC	[1585]	Ran GTPase binding	24 bp	Coding
Indel-046_R	TTTGTGCTTTGGCACCATTA				
Indel-047_F	GCGCCTTTCTTTCACAACTC	[1596]	YABBy-like transcription factor	18 bp	5′ non-coding
Indel-047_R	AACAAAGCTGTTCGGAAGGA				
Indel-048_F	CTCCACATTCTTATCCTCAGATCTG	[3076]	Omega-3 fatty acid desaturase	9 bp	Coding
Indel-048_R	CTCATTGACCTCCATGGATCC				

**Table 3:** **Polymorphic information of 16 InDel markers among six botanical types of cultivated peanut**.

**Markers**	**Distribution of polymorphic InDels marker**						**PCR product**	**PIC**
	***Fastigiata***	***hypogaea***	***vulgaris***	***peruviana***	***hirsuta***	***aequatoriana***		
InDel-03					√		440	0.0169
InDel-04	√	√			√		310	0.0830
InDel-05	√				√	√	420	0.0666
InDel-07		√					430	0.0169
InDel-011		√					470	0.0169
InDel-016	√	√	√		√	√	320	0.5288
InDel-017		√		√	√	√	320	0.1151
InDel-018	√	√	√	√	√		470	0.5960
InDel-020		√			√		390	0.0336
InDel-029	√	√					300	0.0336
InDel-030	√				√	√	240	0.0502
InDel-032		√				√	400	0.2232
InDel-033					√	√	300	0.0336
InDel-039					√	√	200	0.0666
InDel-042					√	√	250	0.1467
InDel-046	√				√	√	300	0.1310
Total	7	9	2	2	12	9		

**Table 4:** **Number of alleles, PIC of different botanical types based on the InDel markers**.

**Botanical type**	**No. of accessions**	**Alleles**	**PIC**
*fastigiata*	34	7	0.1763
*hypogaea*	55	9	0.1809
*vulgaris*	12	2	0.3056
*peruviana*	3	2	0.5556
*hirsuta*	7	12	0.6597
*aequatoriana*	7	9	0.5341
Total	118	16	0.1457

### The genetic relationships revealed by InDel markers among 6 botanical varieties

**Figure 3:**
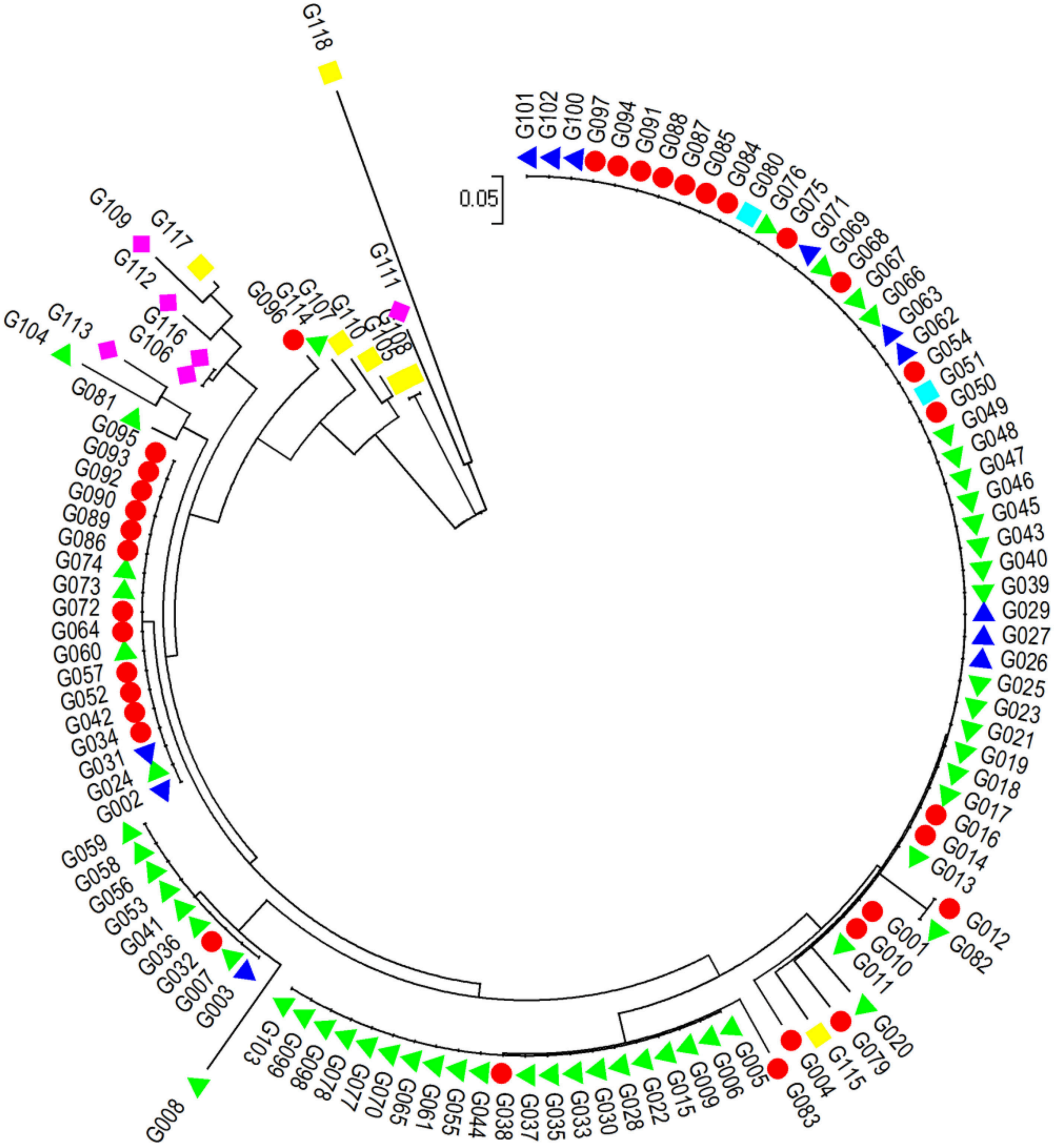
**Dengrogram of 118 accessions of six botanical varieties of cultivated peanuts based on 16 polymorphism Indel makers with a neighbor-joining (NJ) algorithm method**. 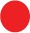 - var. *fastigiata*, 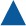 - var. *vulgaris*, 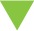 -*var. hypogaea*, 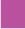 - var. *aequatoriana*, 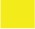 - var. *hirsuta*, 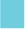 - *var. peruviana*.

A neighbor-joining (NJ) algorithm method assigned the 118 accessions into four major basic groups and some small clusters. Cluster 1 consists of 51 accessions from G101 to G004 (Figure 3). This is a complex cluster, in which var. *fastigiata;* var. *vulgaris; var. hypogaea var. peruviana* were included. Cluster 2 has all 20 *var. hypogaea* accessions (from G005 to G103) plus two var. *fastigiata* G038 and G083. In cluster 3, eight of 10 accessions are *var. hypogaea* (G008 to G059). Cluster 4 contains 12 var. *fastigiata* accessions, 4 var. *hypogaea* accessions (G024, G060, G073, and G074), and 2 var. *vulgaris* accessions (G002 and G031). The rest of 15 accessions formed small clusters. They are mainly var. *aequatoriana* lines and var. *hirsuta* lines and have longest genetic distances to other 4 botanical varieties. The results from this analysis are consistent with the PIC values among different botanical varieties.

### Marker–trait correlation

Five markers, InDel-016, InDel-018, InDel-032, InDel-042, and InDel-046, were identified by single marker analysis to be significantly correlated to tomato spotted wilt virus (TSWV) and leaf spot resistance. Among them, three markers (InDel-032, InDel-042, and InDel-046) were associated to both TSWV and leaf spot resistance, but InDel-018 and 046 were only for leaf spot (Table 4). These markers were designed from conserved sequences of functional genes that were associated with alkaline alpha galactosidase, arachin *Ahy-3*, electron transporter/metal ion, dihydroxy-acid dehydratase, and ran GTPase binding, respectively. InDel-018 and InDel-046 were from the coding region, while InDel-016, InDel-032, and InDel-042 were from non-coding region (Table 2).

In general, the accessions carrying the alleles of the markers had a low leaf spot rate or low percentages of TSWV incidents (Table 5). For example, 43 accessions with InDel-018 alleles had an average of 2.9 leaf spot rate while 75 accessions without the alleles had an average of 4.1 (Table 5). Similar results were observed for TSWV, in which the accessions carrying the alleles of InDel-032 showed a low disease incident (10.7%) compared to the accessions that are lacking of the alleles (46.1%) (Table 5).

**Table 5:** **Significance (*P*-value) of associations between the InDel makers and the targeted traits**.

**Marker**	**Leaf spot**				**TSWV**			
	***P*-value**	**Mean of rate**	**Number of lines**	**Genotype**	***P*-value**	**Mean of rate**	**Number of lines**	**Genotype**
InDel-016	0.0099	3.9	81	+	−	−	−	−
		3.1	37	−		−	−	−
InDel-018	< 0.0001	4.1	75	+	−	−	−	−
		2.9	43	−		−	−	−
InDel-032	< 0.0001	4.1	104	+	< 0.0001	46.1%	104	+
		0.28	14	−		10.7%	14	−
InDel-042	< 0.0001	4.0	109	+	< 0.0001	44.5%	109	+
		0	9	−		11.1%	9	−
InDel-046	< 0.0001	3.9	110	+	0.0053	43.5%	110	+
		0.7	8	−		20%	8	−

## Discussion

Difference in genetic pattern or polymorphism is a main criterion to evaluate the potential functionality of DNA molecular markers. In the present study, the polymorphism of the InDel markers was 33.3%, which was higher than some markers that have been previously reported as to RAPD marker (6.6%) by Subramanian et al. (2000); AFLP marker (3.6%) by He and Prakash (1997); EST-SSR marker (10.4%) by Liang et al. (2009); SSR marker (14.5%) by Zhao et al. (2012) but was lower than Start Codon Targeted polymorphism (SCoT) marker (38.2%) as reported by Xiong et al. (2011) (Table 6). Among the reports, the numbers of accessions evaluated were much less than the 118 accessions used in this study. In general, the larger the number of accessions with diverse genetic background the higher the accuracy of estimated polymorphism associated with a particular trait. Therefore, our reported polymorphism for the InDel markers in this study can be useful in peanut breeding programs.

**Table 6:** **Comparisons of the polymorphism of various molecular markers developed in the previous reports**.

**Marker**	**No. of markers tested**	**Polymorphic markers**	**Polymorphism rate (%)**	**No. of accessions tested**	**No. of botanical types**	**References**
RADP	408	27	6.6	70	4	Subramanian et al., 2000
AFLP	111	4	3.6	6	3	He and Prakash, 1997
EST-SSR	251	26	10.4	22	4	Liang et al., 2009
SSR	9274	1343	14.5	8	Var.	Zhao et al., 2012
ScoT	157	60	38.2	20	4	Xiong et al., 2011
InDel	48	16	33.3	118	6	Present study

Germplasm resources provide fundamental materials for peanut genetic improvement, and the study of genetic diversity on cultivated peanut will enhance the utilization of peanut genetic resources. Genetic diversity of six botanical types of cultivated peanuts has been extensively investigated using molecular markers. Based on SSR markers, Jiang et al. (2010) demonstrated that the accessions of *fastigiata* and *hypogaea* were more diversified than other botanical types. The genetic diversity of 72 accessions of the U.S. mini core was estimated using 67 SSR primer pairs and the results indicated that the PIC of SSR markers ranged from 0.063 to 0.918 and the gene diversity ranged from 0.027 to 0.50 (Kottapalli et al., 2007). In the present study, PICs varied from 0.176 for *fastigiata* var. to 0.660 for *hirsuta* var., and *hirsuta* var., *peruviana* var., and *aequatoriana* var. have higher genetic diversity than the other types, indicating that, like other molecular markers, InDel markers can be used for evaluation of genetic diversity for peanuts. Cluster analysis showed that *hirsuta* var. and *aequatoriana* var. have longest genetic distances from the other four types, indicating that *hirsuta* var. and *aequatoriana* var. have higher genetic diversity than the other types.

Unlike the QTL that using biparental RIL (Recombinant Inbred Lines) mapping populations to link markers with target traits, the identified marker trait association in present cannot validated in different backgrounds, but in our another apparel association mapping study we have extensively evaluated leaf spot and TSWV resistances for the U.S. mini-core collection and mapped three SSR markers named “pPGPseq2D12B,” “pPGSseq19B1,” and “TC04F12,” to be associated both with leaf spot and TSWV resistances. The marker “TC20B05” can explain 15% phenotypical variation of leaf spot resistance.

Regarding application of MAS in peanut, there are only two molecular markers currently being utilized in breeding programs: nematode resistance and high oleic seed chemistry. Chu et al. (2011) demonstrated that a tremendous reduction in the amount of time (at least 3-fold) for plant selection was achieved with MAS to pyramid nematode resistance with high oleic trait in peanut. This recent success is only possible due to the initial discovery of the genetic markers and the development of breeding lines. For example, the identification of high oleic marker was achieved by utilizing different genes in fatty acid biosynthesis for high oleic chemistry in other oil seed crops enabling a straightforward characterization in peanut and discovery of similar functional mutations in breeding populations (Jung et al., 2000; Lopez et al., 2002). Nematode resistance was introgressed from wild species (Simpson and Starr, 2001), and resistant plants were selected based on the availability of molecular markers at the time (Nagy et al., 2010). High Oleic trait resulted from the expression of two recessive genes (Lopez et al., 2001) while nematode resistance was determined to result from the expression of two dominant genes (Garcia et al., 1996). For other traits such as disease resistance or drought tolerance, complex interaction between genetic and environment poses daunting challenge to breeders to select resistant plants. Since InDel markers were developed from sequences of functional genes, they will lay the groundwork for the identification of genes related to superior agronomic traits, provide information on population genetic variations, and identify homologous genes for functional studies. Since InDel markers were found to be associated with leaf spot and TSWV resistance with a higher level of DNA polymorphism compared to other molecular markers, they provide a very useful type of molecular marker to identify other agronomical important traits in peanut.

### Conflict of interest statement

The authors declare that the research was conducted in the absence of any commercial or financial relationships that could be construed as a potential conflict of interest.

## References

[B1] Belamkar V. Selvaraj M. G. Ayers J. L. Payton P. R. Puppala N. Burow M. D.. (2011). A first insight into population structure and linkage disequilibrium in the US peanut minicore collection. Genetica 139, 411–429. 10.1007/s10709-011-9556-2 21442404

[B2] Burow M. D. Simpson C. E. Faries M. W. Starr J. L. Paterson A. H. (2009). Molecular biogeographic study of recently described B- and A-genome Arachis species, also providing new insights into the origins of cultivated peanut. Genome 52, 107–119. 10.1139/G08-094 19234559

[B3] Burow M. D. Simpson C. E. Paterson A. H. Starr J. L. (1996). Identification of peanut (*Arachis hypogaea* L.) RAPD markers diagnostic of root-knot nematode (*Meloidogyne arenaria* (Neal) Chitwood) resistance. Mol. Breeding 2, 369–319. 10.1007/BF00437915

[B4] Chen C. Y. Barkley N. A. Wang M. L. Holbrook C. C. Dang P. M. (2014). Registration of purified accessions for the U.S. peanut mini-core germplasm collection. J. Plant Reg. 8, 77–85. 10.3198/jpr2013.01.0003crg

[B5] Chiteka Z. A. Gorbet D. W. Knauft D. A. Shokes F. M. Kucharek T. A. (1988). Components of resistance to late leaf spot in peanut. Peanut Sci. 15, 76–81. 10.3146/i0095-3679-15-2-9

[B6] Chu Y. Wu C. L. Holbrook C. C. Tillman B. L. Person G. Ozias-Akins P. (2011). Marker-assisted selection to pyramid nematode resistance and the high oleic trait in peanut. Plant Genome 4, 110–117. 10.3835/plantgenome2011.01.0001

[B7] Cuc L. M. Mace E. S. Crouch J. H. Quang V. D. Long T. D. Varshney R. K. (2008). Isolation and characterization of novel microsatellite markers and their application for diversity assessment in cultivated groundnut (*Arachis hypogaea*). BMC Plant Biol. 8:55. 10.1186/1471-2229-8-55 18482440 PMC2416452

[B8] Dang P. M. Chen C. Y. (2013). Modified method for combined DNA and RNA isolation from peanut and other oil seeds. Mol. Biol. Rep. 40, 1563–1568. 10.1007/s11033-012-2204-9 23104473

[B9] Feng S. Wang X. Zhang X. Dang P. M. Holbrook C. C. Culbreath A. K.. (2012). Peanut (*Arachis hypogaea*) expressed sequence tag project: progress and application. Comp. Funct. Genomics 2012:373768. 10.1155/2012/373768 22745594 PMC3382957

[B10] Garcia G. M. Stalker H. T. Shroeder E. Kochert G. (1996). Identification of RAPD, SCAR and RFLP markers tightly linked to nematode resistance genes introgressed from *Arachis cardenasii* to *A. hypogaea*. Genome 39, 836–845. 8890516 10.1139/g96-106

[B11] Gautami B. Foncéka D. Pandey M. K. Moretzsohn M. C. Sujay V. Qin H.. (2012). An international reference consensus genetic map with 897 marker loci based on 11 mapping populations for tetraploid groundnut (*Arachis hypogaea* L.). PLoS ONE 7:e41213. 10.1371/journal.pone.0041213 22815973 PMC3399818

[B12] Guo B. Chen X. Hong Y. Liang X. Dang P. Brenneman T.. (2009). Analysis of gene expression profiles in leaf tissues of cultivated peanuts and development of EST- SSR markers and gene discovery. Int. J. Plant Genomics 2009:715605. 10.1155/2009/715605 19584933 PMC2703745

[B13] He G. H. Prakash C. S. (1997). Identification of polymorphic DNA markers in cultivated peanut (*Arachis hypogaea* L.). Euphytica 97, 143–149. 10.1023/A:1002949813052

[B14] Hong Y. Chen X. Liang X. Liu H. Zhou G. Li S.. (2010). A SSR-based composite genetic linkage map for the cultivated peanut (*Arachis hypogaea* L.) genome. BMC Plant Biol. 10:17. 10.1186/1471-2229-10-17 20105299 PMC2835713

[B15] Jansen R. C. Stam P. (1994). High resolution of quantitative traits into multiple loci via interval mapping. Genetics 136, 1447–1455. 8013917 10.1093/genetics/136.4.1447 PMC1205923

[B16] Jiang H. F. Ren X. P. Zhang X. J. Huang J. Q. Lei Y. Yan L. Y. (2010). Comparison of genetic diversity between peanut mini core collections from China and ICRISAT by SSR markers. Acta Agr. Sin. 36, 1084-1091. 10.3724/SP.J.1006.2010.01084

[B17] Jiang H. Huang L. Ren X. Chen Y. Zhou X. Xia Y.. (2014). Diversity characterization and association analysis of agronomic traits in a Chinese peanut (*Arachis hypogaea* L.) mini-core collection. J. Integr. Plant Biol. 56, 159–169. 10.1111/jipb.12132 24237710

[B18] Jung S. Swift D. Sengoku E. Patel M. Teule F. Powell G.. (2000). The high oleate trait in the cultivated peanut (*Arachis hypogaea* L.). Isolation and characterization of two genes encoding microsomal oleoyl-PC desaturases. Mol. Gen. Genet. 263, 796–805. 10.1007/s004380000244 10905347

[B19] Koilkonda P. Sato S. Tabata S. Shirasawa K. Hirakawa H. Sakai H.. (2012). Large-scale development of expressed sequence tag-derived simple sequence repeat markers and diversity analysis in Arachis spp. Mol. Breeding 30, 125–138. 10.1007/s11032-011-9604-8 22707912 PMC3362703

[B20] Kottapalli K. R. Burow M. D. Burow G. Burke J. Puppala N. (2007). Molecular characterization of the US peanut mini core collection using microsatellite markers. Crop Sci. 47, 1718–1727. 10.2135/cropsci2006.06.0407

[B21] Liang X. Chen X. Hong Y. Liu H. Zhou G. Li S.. (2009). Utility of EST-derived SSR in cultivated peanut (*Arachis hypogaea* L.) and Arachis wild species. BMC Plant Biol. 9:35. 10.1186/1471-2229-9-35 19309524 PMC2678122

[B22] Liu B. Wang Y. Zhai W. Deng J. Wang H. Cui Y.. (2013). Development of InDel markers for Brassica rapa based on whole-genome re-sequencing. Theor. Appl. Genet. 126, 231–239. 10.1007/s00122-012-1976-6 22972202

[B23] Lopez Y. Nadaf H. L. Smith O. D. Simpson C. E. Fritz A. K. (2002). Expressed variants of Delta(12)-fatty acid desaturase for the high oleate trait in spanish market-type peanut lines. Mol. Breeding 9, 183–190. 10.1023/A:1019767825486

[B24] Lopez Y. Smith O. D. Senseman S. A. Rooney W. L. (2001). Genetic factors influencing high oleic acid content in Spanish market-type peanut cultivars. Crop Sci. 41, 51–56. 10.2135/cropsci2001.41151x

[B25] Lv H. H. Yang L. M. Kang J. G. Wang Q. B. Wang X. W. Fang Z. Y. (2013). Development of InDel markers linked to Fusarium wilt resistance in cabbage. Mol. Breeding 32, 961–967. 10.1007/s11032-013-9925-x

[B26] Moretzsohn M. C. Gouvea E. G. Inglis P. W. Leal-Bertioli S. C. Valls J. F. Bertioli D. J. (2013). A study of the relationships of cultivated peanut (*Arachis hypogaea*) and its most closely related wild species using intron sequences and microsatellite markers. Ann. Bot. 111, 113–126. 10.1093/aob/mcs237 23131301 PMC3523650

[B27] Mukri G. Nadaf H. L. Bhat R. S. Gowda M. V. C. Upadhyaya H. D. Sujay V. (2012). Phenotypic and molecular dissection of ICRISAT mini core collection of peanut (*Arachis hypogaea* L.) for high oleic acid. Plant Breeding 131, 418–422. 10.1111/j.1439-0523.2012.01970.x

[B28] Nagy E. D. Chu Y. Guo Y. Khanal S. Tang S. Li Y. (2010). Recombination is suppressed in an alien introgression in peanut harboring Rma, a dominant root-knot nematode resistance gene. Mol. Breed. 26, 357–370. 10.1007/s11032-010-9430-4

[B29] Nagy E. D. Guo Y. Tang S. Bowers J. E. Okashah R. A. Taylor C. A.. (2012). A high-density genetic map of *Arachis duranensis*, a diploid ancestor of cultivated peanut. BMC Genomics 13:469. 10.1186/1471-2164-13-469 22967170 PMC3542255

[B30] Qin H. D. Feng S. P. Chen C. Guo Y. Knapp S. Culbreath A.. (2012). An integrate genetic linkage map of cultivated peanut (*Arachis hypogaea* L.) constructed from two RIL populations. Theor. Appl. Genet. 124, 653–664. 10.1007/s00122-011-1737-y 22072100

[B31] Raina S. N. Rani V. Kojima T. Ogihara Y. Singh K. P. Devarumath R. M. (2001). RAPD and ISSR fingerprints as useful genetic markers for analysis of genetic diversity, varietal identification, and phylogenetic relationships in peanut (*Arachis hypogaea)* cultivars and wild species. Genome 44, 763–772. 10.1139/gen-44-5-763 11681599

[B32] Ravi K. Vadez V. Isobe S. Mir R. R. Guo Y. Nigam S. N.. (2011). Identification of several small main-effect QTLs and a large number of epistatic QTLs for drought tolerance related traits in groundnut (*Arachis hypogaea* L.). Theor. Appl. Genet. 122, 1119–1132. 10.1007/s00122-010-1517-0 21191568 PMC3057011

[B33] Ren X. P. Zhang X. J. Liao B. S. Lei Y. Huang J. Q. Chen Y. N. (2010). Analysis of genetic diversity in ICRISAT mini core collection of peanut (*Arachis hypogaea* L.) by SSR markers. Sci. Agr. Sin. 43, 2848–2858.

[B34] Rockah-Shmuel L. Tóth-Petróczy Á. Sela A. Wurtzel O. Sorek R. Tawfik D. S. (2013). Correlated occurrence and bypass of frame-shifting insertion-deletions (InDels) to give functional proteins. PLoS Genet 9:e1003882. 10.1371/journal.pgen.1003882 24204297 PMC3812077

[B35] Saitou N. Nei M. (1987). The neighbor-joining method: a new method for reconstructing phylogenetic trees. Mol. Biol. Evol. 4, 406–425. 3447015 10.1093/oxfordjournals.molbev.a040454

[B36] SAS Institute (2009). SAS User's Guide: Statistics, Version 9.2. Cary, NC: SAS Inst.

[B37] Shirasawa K. Bertioli D. J. Varshney R. K. Moretzsohn M. C. Leal-Bertioli S. C. Thudi M.. (2013). Integrated consensus map of cultivated peanut and wild relatives reveals structures of the A and B genomes of Arachis and divergence of the legume genomes. DNA Res. 20, 173–184. 10.1093/dnares/dss042 23315685 PMC3628447

[B38] Simpson C. E. Starr J. L. (2001). Registration of ‘COAN’ peanut. Crop Sci. 41, 918 10.2135/cropsci2001.413918x

[B39] Subramanian V. Gurtu S. Nageswara Rao R. C. Nigam S. N. (2000). Identification of DNA polymorphism in cultivated groundnut using random amplified polymorphic DNA (RAPD) assay. Genome 43, 656–660. 10.1139/g00-034 10984178

[B40] Sujay V. Gowda M. V. Pandey M. K. Bhat R. S. Khedikar Y. P. Nadaf H. L.. (2012). Quantitative trait locus analysis and construction of consensus genetic map for foliar disease resistance based on two recombinant inbred line populations in cultivated groundnut (*Arachis hypogaea* L.). Mol. Breeding 30, 773–788. 10.1007/s11032-011-9661-z 22924018 PMC3410029

[B41] Tamura K. Dudley J. Nei M. Kumar S. (2007). MEGA4: molecular evolutionary genetics analysis (MEGA) software version 4.0. Mol. Biol. Evol. 24, 1596–1599. 10.1093/molbev/msm092 17488738

[B42] Tautz D. (1989). Hypervariability of simple sequences as a general source for polymorphic DNA markers. Nucleic Acids Res. 17, 6463–6471. 10.1093/nar/17.16.6463 2780284 PMC318341

[B43] Tillman B. L. Gorbet D. W. Andersen P. C. (2007). Influence of planting date on yield and tomato spotted wilt of runner market type peanut. Peanut Sci. 34, 79–84. 10.3146/0095-3679(2007)34[79:IOPDOY]2.0.CO;2

[B44] Upadhyaya H. D. Mukri G. Nadaf H. L. Singh S. (2012). Variability and stability analysis for nutritional traits in the mini core collection of peanut. Crop Sci. 52, 168–178. 10.2135/cropsci2011.05.0248

[B45] Varshney R. K. Bertioli D. J. Moretzsohn M. C. Vadez V. Krishnamurthy L. Aruna R.. (2009). The first SSR-based genetic linkage map for cultivated groundnut (*Arachis hypogaea* L.). Theor. Appl. Genet. 118, 729–739. 10.1007/s00122-008-0933-x 19048225

[B46] Vos P. Hogers R. Bleeker M. Reijans M. van de Lee T. Hornes M.. (1995). AFLP: a new technique for DNA fingerprinting. Nucl. Acids Res. 23, 4407–4414. 10.1093/nar/23.21.4407 7501463 PMC307397

[B47] Wang H. Penmetsa R. V. Yuan M. Gong L. Zhao Y. Guo B.. (2012). Development and characterization of BAC-end sequence derived SSRs, and their incorporation into a new higher density genetic map for cultivated peanut (*Arachis hypogaea* L.). BMC Plant Biol. 12:10. 10.1186/1471-2229-12-10 22260238 PMC3298471

[B48] Wang M. L. Chen C. Y. Tonnis B. Barkley N. A. Pinnow D. L. Pittman R. N.. (2013). Oil, fatty acid, flavonoid, and resveratrol content variability and FAD2A functional SNP genotypes in the U.S. peanut mini-core collection. J. Agr. Food Chem. 61, 2875–2882. 10.1021/jf305208e 23379758

[B49] Wang M. L. Sukumaran S. Barkley N. A. Chen Z. Chen C. Y. Guo B.. (2011). Population structure and marker-trait association analysis of the US peanut (*Arachis hypogaea* L.) mini-core collection. Theor. Appl. Genet. 123, 1307–1317. 10.1007/s00122-011-1668-7 21822942

[B50] Williams J. G. Kubelik A. R. Livak K. J. Rafalski J. A. Tingey S. V. (1990). DNA polymorphisms amplified by arbitrary primers are useful as genetic markers. Nucleic Acids Res. 18, 6531–6535. 10.1093/nar/18.22.6531 1979162 PMC332606

[B51] Wu K. Yang M. Liu H. Tao Y. Mei J. Zhao Y. (2014). Genetic analysis and molecular characterization of Chinese sesame (*Sesamum indicum* L.) cultivars using insertion-deletion (InDel) and simple sequence repeat (SSR) markers. BMC Genet 15:35. 10.1186/1471-2156-15-35 24641723 PMC4234512

[B52] Xiong F. Zhong R. Han Z. Jiang J. He L. Zhuang W.. (2011). Start codon targeted polymorphism for evaluation of functional genetic variation and relationships in cultivated peanut (*Arachis hypogaea* L.) genotypes. Mol. Biol. Rep. 38, 3487–3494. 10.1007/s11033-010-0459-6 21104441

[B53] Yamaki S. Ohyanagi H. Yamasaki M. Eiguchi M. Miyabayashi T. Kubo T.. (2013). Development of INDEL markers to discriminate all genome types rapidly in the genus Oryza. Breeding Sci. 63, 246–254. 10.1270/jsbbs.63.246 24273419 PMC3770551

[B54] Zhang J. Liang S. Duan J. Wang J. Chen S. Cheng Z.. (2012). De novo assembly and characterization of the transcriptome during seed development, and generation of genic-SSR markers in peanut (*Arachis hypogaea* L.). BMC Genomics 13:90. 10.1186/1471-2164-13-90 22409576 PMC3350410

[B55] Zhao Y. L. Prakash C. S. He G. (2012). Characterization and compilation of polymorphic simple sequence repeat (SSR) markers of peanut from public database. BMC Res. Notes 5, 362–369. 10.1186/1756-0500-5-362 22818284 PMC3500262

[B56] Zietkiewicz E. Rafalski A. Labuda D. (1994). Genome fingerprinting by simple sequence repeat (SSR)-anchored polymerase chain reaction amplification. Genomics 20, 176–183. 10.1006/geno.1994.1151 8020964

